# Effects of Antecedent and Consequence Secondary Targets on Intraverbal Teaching

**DOI:** 10.1007/s40616-026-00229-1

**Published:** 2026-06-08

**Authors:** Julian Cifuentes, Manish Goyal, Lesley A. Shawler

**Affiliations:** School of Psychological and Behavioral Sciences, Southern Illinois University, Carbondale, Carbondale, IL USA

**Keywords:** Instructive feedback, Secondary targets, Preference, Efficiency

## Abstract

Children diagnosed with autism spectrum disorder (ASD) may have difficulty with social interactions and communication. Thus, identifying procedures that are efficient and effective at improving communication is important. Instructive feedback (IF) is a procedure that has been found to increase communication for individuals with developmental disabilities by teaching a primary target (PT) and embedding a secondary target (ST) within the learning trial. However, different procedural configurations exist across the IF literature, which may influence its efficacy and efficiency. The purpose of this study was to compare the delivery of antecedent and consequence ST when teaching different tacts and intraverbals to a child diagnosed with ASD across multiple phases and stimulus sets. In phase 1, we compared teaching antecedent and consequence ST to no ST and found that incorporating ST resulted in better acquisition of ST compared to no ST. In phases 2 and 3, we compared only antecedent and consequence ST and showed that both conditions led to the acquisition of intraverbals; however, acquisition rates differed across conditions. Finally, in phase 3, we assessed the participant’s allocation of responding between the different ST types in a concurrent-link arrangement. The participant allocated responding more to the consequence condition, which was generally more efficient at producing ST acquisition in phases 2 and 3. Possible explanations for differences in acquisition related to when ST were delivered are discussed, as well as areas for future research.

**Supplementary Information:**

The online version contains supplementary material available at 10.1007/s40616-026-00229-1.

Teaching intraverbals and tacts using discrete-trial instruction (DTI; Smith, 2001) is a common goal in intervention when teaching children with autism spectrum disorder (ASD). However, previous reports have suggested that, in some contexts, DTI may occasion more rote responding and require greater instructional resources (Delfs et al., 2014; Frampton & Shillingsburg, 2020). Instructive feedback (IF) is one method that was developed to improve the efficiency and efficacy of instructional procedures (e.g., DTI; Reichow & Wolery, 2011; Werts et al., 1995) by providing additional feedback within a learning trial. For example, while teaching a primary target (PT) response (e.g., tacting “dog” in the presence of a picture of a dog), a secondary target (ST, e.g., “it is an animal”) is added to the discrete trial with no response requirement to the ST. As a result, the learner may acquire both targets with direct teaching of the PT only. Studies have shown the efficacy of IF when teaching intraverbal responses (e.g., Carroll & Kodak, 2015; Frampton & Shillingsburg, 2020), play behavior (e.g., Grow et al., 2017; Morton et al., 2023), tacting (e.g., Delmolino et al., 2013; Gavidia et al., 2022), and listener responding (e.g., Jimenez-Gomez et al., 2021) to individuals with ASD. Moreover, some studies have shown increased efficiency (i.e., trials to criterion, instructional time) when using IF to teach tacts or intraverbals compared to DTI procedures (e.g., Nottingham et al., 2017; Vladescu & Kodak, 2013).

Several IF procedural variations have been tested with mixed outcomes (e.g., Haq et al., 2017; Tullis et al., 2018). In some of these variations, the ST can be arranged at the beginning (as an antecedent) or at the end (as a consequence) of a learning trial. For example, the ST (e.g., “It produces milk!”) can be presented either before presenting the picture of a cow, or with praise after the correct tact response is given (e.g., “Great job! It produces milk”). However, few studies have evaluated the effects of different IF procedural configurations (e.g., antecedent or consequence) on the ST acquisition across verbal operants (c.f. Vladescu & Kodak, 2013; Wolery et al., 2000). Thus, it is unclear whether the ST locus within a learning trial influences its acquisition.

Wolery et al. (2000) compared the efficiency of presenting the ST as an antecedent and as a consequence during tact teaching with three children diagnosed with developmental disabilities. In each trial, target words (i.e., ST) unrelated to the target stimuli (i.e., PT) were presented either in the antecedent or consequence portion of the trial within an adapted alternating treatments design. Efficiency was measured as the number of sessions and the percentage of errors until mastery criteria were reached for both PT and ST. Overall, Wolery et al. found minimal differences in efficiency between conditions when delivering the ST, suggesting the ST can be provided in either the antecedent or consequence portion of the trial. Vladescu and Kodak (2013) extended Wolery et al. (2000) with children with ASD and similarly found minimal differences in ST acquisition (as tacts and intraverbals) between antecedent and consequence conditions. However, notably, they measured efficiency as overall time per target learned rather than the number of sessions or errors.

Research has found that IF efficacy may depend on the learner’s response (i.e., observing and/or echoic responses; Haq et al., 2017) or demand characteristics (i.e., history of reinforcement with similar trial structures; Reichow & Wolery, 2011). Haq et al. (2017) examined whether participant behavior and feedback configuration improved the ST acquisition with two children diagnosed with Fragile X syndrome and ASD. Depending on performance and learner characteristics, participants were prompted to emit an observing response toward the discriminative stimulus (S^D^) (i.e., looking at the picture for 1 s) or echo the ST. Results suggest a heterogeneous effect of specific learner characteristics (e.g., attending and/or generalized vocal imitation) and ST locus (antecedent or consequence) on ST acquisition. However, researchers have noted the absence of within-subject replications (Haq et al., 2017; Tullis et al., 2018; Vladescu & Kodak, 2013), which has contributed to the lack of clear findings.

The purpose of this study was to extend and build on past research comparing the delivery of antecedent and consequence ST by evaluating the efficacy and efficiency of procedures for one child with ASD during tact and intraverbal teaching using within-subject replication. Differences between antecedent and consequence ST were assessed, addressing three limitations from previous research, resulting in the following research questions:

1. What are the differences between antecedent- and consequence-ST on the acquisition of both PT and ST across within-subject replications?
2. How do antecedent- and consequence-ST differentially affect the transfer-of-stimulus control from tacts to intraverbals?, and
3. Do children with ASD prefer the delivery of one type of ST over another?

By building on past studies (e.g., Vladescu & Kodak, 2013; Wolery et al., 2000), we sought to better understand the processes that may be involved in each ST configuration, which could help to preemptively prescribe one procedure over another depending on certain characteristics observed.

## Method

### Participant and Setting

Harrison was a White 4-year-old male diagnosed with level 1 ASD who communicated in approximately 3–6-word phrases. Harrison was 3 years old at the start of the study but turned 4 during phase 2; he participated in all three phases. At the time of the study, Harrison had received applied behavior analytic services for approximately 6 months, for 4 hours a week. At the onset of the study, Harrison scored 114 points on the Verbal Behavior Milestones Assessment and Placement Program (VB-MAPP; Sundberg, 2008), scoring primarily in the level 2 (18–30 months) range. Harrison scored 69 on the intraverbal subtest (Sundberg, 2008), which examines responding to various questions in sequence of difficulty, from fill-in-the-blank responses to answering multiple-part questions. Further, a language sample completed around the start of the study reported Harrison’s mean length of utterance at 4.25–4.77 words. The Preschool Language Scale-5 (Zimmerman et al., 2011), a norm-referenced assessment measuring speaker and listener skills for children between birth and 7 years 11 months, was completed 5 months before the start of the study. Harrison’s scores on the Preschool Language Scale-5 auditory comprehension, expressive communication, and combined standard scores were 89, 74, and 80, respectively (*M* = 100, *SD =* 15). According to the assessment scores, Harrison’s auditory comprehension score was within normal limits, while his expressive communication score was below average, indicating the need for intervention for speaker behavior.

All sessions occurred in a small therapy room measuring 2.1 m x 1.8 m at a midwestern university-based autism clinic. We conducted two sessions per week for 23 weeks (about 5.5 months) for all three phases for approximately 60–120 min each.

### Materials and Stimuli

Each room contained a table, chairs, preferred items selected by Harrison each day (e.g., cars, blocks), instructional materials (e.g., Webber® cards), and session materials (e.g., clipboards, data sheets, timers, and pens). Stimulus cards were selected from the Webber® cards set and were 8.25 cm x 10.79 cm pictures printed on a white background. For phase 1, profession pictures were selected (e.g., a doctor) and divided into three sets (antecedent, consequence, and PT only). For phase 2, house item pictures were selected (e.g., broom) across two sets (antecedent and consequence). For phase 3, everyday items were selected (e.g., umbrella) and distributed across two sets (antecedent and consequence). Each set consisted of five targets related to the specific category.

### Response Measurement

The dependent variables for all phases were the percentage of correct independent responses for ST, PT, and intraverbals, and the number of trials-to-criterion across the antecedent, consequence, and PT-only conditions. A correct independent response was defined as Harrison emitting the vocal response corresponding to the S^D^ within 5 s. Trials-to-criterion were defined as the number of trials for Harrison to meet the mastery criteria for each phase. During phase 1, sessions consisted of 5-trial blocks with mastery criteria for the PT at three consecutive blocks at 80% or more (4/5) independent correct responses. Mastery criteria for the ST were one block during probes at 80% or more (4/5) independent correct responses. During phases 2 and 3, sessions consisted of 15-trial blocks with mastery criteria for the PT and ST at three consecutive blocks at 80% or more (12/15) independent correct responses. Mastery for the intraverbal responses was one block at 80% or more. Intraverbal responses were considered the terminal goal given that responding would occur in the absence of nonverbal stimuli (pictures). Thus, in phase 2, we used a nonparallel stopping criterion for the ST, meaning we ceased teaching in the antecedent condition before observing the ST mastery criterion, as mastery of the optimal response (i.e., intraverbals) was observed.

### Interobserver Agreement (IOA) and Procedural Fidelity

A second, independent observer collected data, and trial-by-trial IOA was calculated across conditions for each phase. Trial-by-trial IOA was calculated by dividing the number of trials with agreements by the total number of trials multiplied by 100 for each condition. An agreement was defined as both observers recording the same response for the same trial, scored as a 1, and disagreements were scored as a 0.

IOA for phase 1 was collected for approximately 46.6% of trial blocks across all conditions. IOA was high for the antecedent (*M* = 94.6%, range: 86.7–100%), consequence (*M =* 91.7%, range: 86.7–100%), and the PT only (*M =* 100%, range: 93.3–100%) conditions. IOA was collected for approximately 44.4% of trial blocks for all conditions for phase 2. IOA was high for the antecedent condition for both ST probes (*M* = 98.2%, range: 97–100%) and intervention (*M =* 100%), and for the consequence condition for the ST probes (*M =* 100%), and intervention (*M* = 96.6%, range: 90–100%). IOA was collected for 100% of trial blocks across all conditions for phase 3. In the antecedent condition, IOA was high for probes (*M =* 98.5%, range: 93.3–100%) and intervention (*M =* 100%); and were high for the consequence condition probes (*M* = 100%) and intervention (*M* = 99%, range: 93.33–100%).

Procedural fidelity was assessed by a second independent observer during 55.5% of sessions in phase 2 and 45.4% of sessions in phase 3. During each intervention session, the observer recorded whether the experimenter (a) had materials prepared, (b) ensured client was orienting to the clinician, (c) used the correct color-correlated stimuli based on the condition in effect, (d) used various instructions for the PT, (e) used various instructions for the ST, (f) implemented the correct ST locus in the instruction based on the specific condition, (g) delivered verbal praise and specific reinforcement for correct responses, and (h) delivered the error correction procedure for incorrect responses. During the baseline and probe sessions, steps a-e were scored, but the experimenter was also scored as to the absence of feedback provided following Harrison’s responses. Correct implementations were scored as 1, and incorrect implementations were scored as 0. Procedural fidelity was calculated per session as a percentage by dividing the sum of correct implementations by the total number of events multiplied by 100. For phase 2, procedural fidelity for the antecedent condition was a mean of 91% (range: 69–100%), and a mean of 100% for the consequence condition. For phase 3, the mean procedural fidelity for the antecedent condition was 100% and a mean of 93% (range: 86–100%) for the consequence condition.

### Experimental Design

For all phases, an adapted alternating treatments design (Sindelar et al., 1985) was used to compare the effects of antecedent ST, consequence ST, and PT only (phase 1 only) on the levels of correct ST and intraverbal responses. All intervention conditions were randomly alternated with no more than two consecutive presentations of the same condition. For each condition, targets were equally distributed across sets based on Cariveau et al. (2020) guidelines using a logical analysis to control the number of syllables and words for all targets (Table 1).

**Table 1:** Logical analysis for professions for phase 1 – primary and secondary targets

	Targets		Number syllables		Number words	
Sets	Primary (PT)	Secondary (ST)	PT	ST	PT	ST
Set 1	Vet	Cure animals	1	4	1	2
	Farmer	Grow food	2	2	1	2
	Pastor	Guide church	2	2	1	2
	Dentist	Fix teeth	2	2	1	2
	Astronaut	study space	3	3	1	2
Set 2	Plumber	Fix pipes	2	2	1	2
	Doctor	Cure sickness	2	3	1	2
	Teacher	Teach children	2	3	1	2
	Soldier	Protect people	2	4	1	2
	Athlete	Play sports	2	2	1	2
Set 3	Cop	Prevent crime	1	3	1	2
	Mailman	Deliver mail	2	4	1	2
	Lawyer	Protect laws	2	3	1	2
	Actor	act play	2	2	1	2
	Mechanic	Fix car	3	2	1	2
	Total	Set 1	10	13	5	10
		Set 2	10	14	5	10
		Set 3	10	14	5	10

Logical analysis for profession targets. Targets are distributed across sets using recommendations by Cariveau et al. (2020) according to the number of syllables and the number of words

### Procedure

At the beginning of the study, a list of items or people (PT) and related functions (ST) across all three categories was probed to identify unknown targets. Targets that were incorrect for both the PT and ST were selected for phases 1 and 2 and distributed across conditions using a logical analysis. For phase 3, Harrison scored correctly on two targets when testing, and these targets were counterbalanced across both conditions.

Before each session and periodically during breaks, the experimenter asked Harrison what he wanted to play with during work time. Harrison then selected two to four toys that were used as reinforcers and delivered during teaching trials on a fixed-ratio 5 schedule (i.e., each target was presented one time). As such, reinforcers varied daily depending on Harrison’s responses.

#### Baseline

**Fig. 1:**
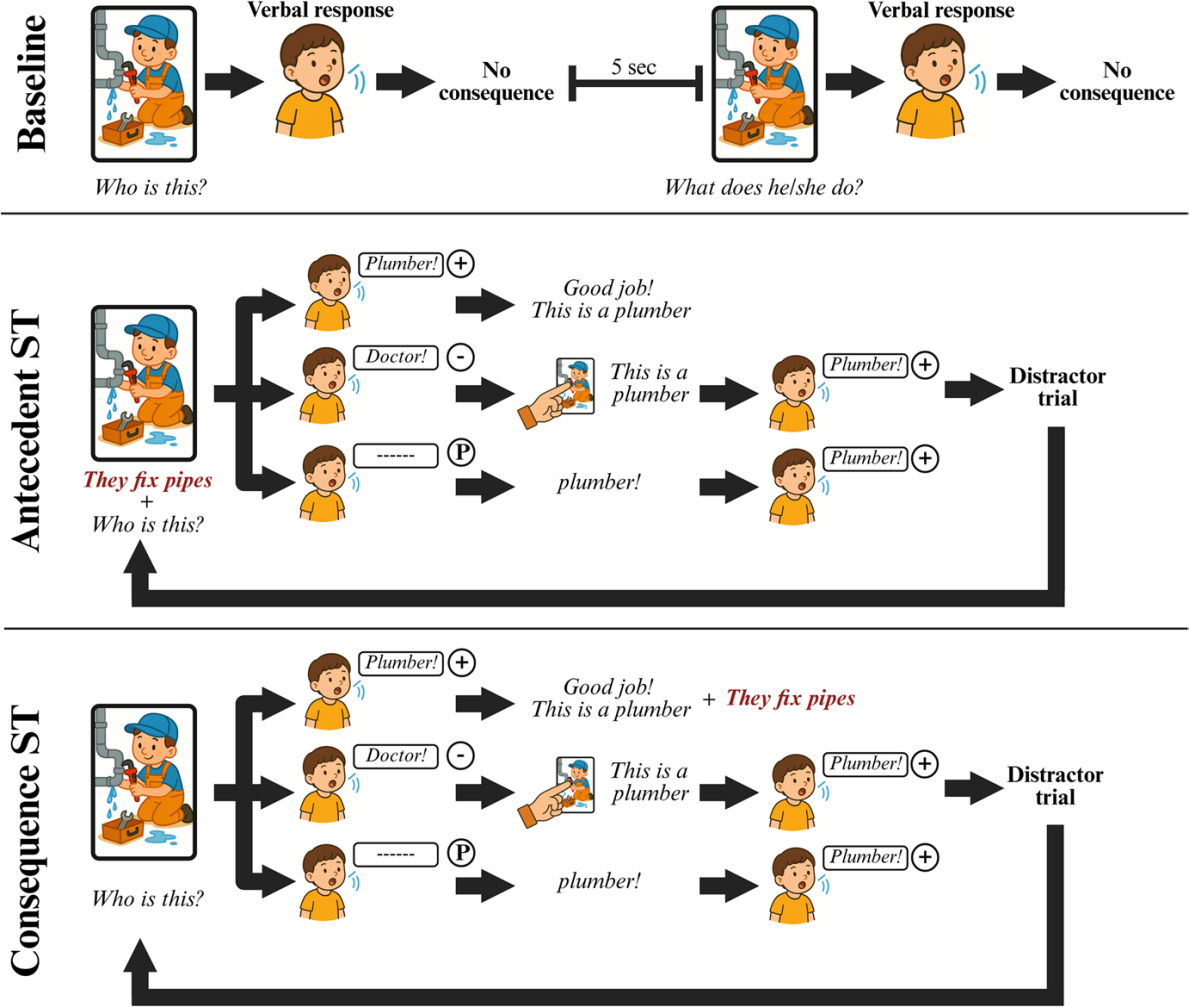
Flow chart depicting the three intervention procedures. *Note: + *correct response,* -* error*, P* prompt; Primary target (PT) depicted with the image card and finger pointing to it; Secondary target (ST) depicted in brown

Figure 1 (top panel) depicts the sequence of steps during baseline. The experimenter presented the PT picture and asked: (a) “Who is this?” or (b) “What do you see?” The experimenter provided no prompts or consequences regardless of the response. After a brief 5-s pause, the experimenter re-presented the picture and asked: (a) “What does it/they do?”, (b) “What does he/she do?”, or (c) “What do you use it for?” for the ST. All targets were interspersed, and the experimenter intermittently provided praise for working (e.g., “Great job sitting in your chair!”) and interspersed maintenance trials to provide Harrison with opportunities to contact reinforcement. No praise or feedback was provided to Harrison for responding to the PT and ST during baseline trials. Once stability in baseline responding occurred for each set, we introduced the teaching conditions.

#### General Tact Teaching (PT)

The experimenter presented the PT as described in baseline. If Harrison responded correctly within 5 s, the experimenter provided behavior-specific praise (e.g., “Good job! This is a plumber”). If Harrison answered incorrectly or did not respond within 5 s, the experimenter implemented error correction by pointing to the picture and modeling the correct vocal response (e.g., “This is a plumber”). The experimenter then removed the picture for 2 s and repeated the trial until a correct response occurred or up to three times. Trials were presented in pseudorandom order.

#### Antecedent Condition

Figure 1 (middle panel) depicts the sequence of steps during the antecedent condition. For every trial, the experimenter first presented a picture and delivered the ST (e.g., “They fix pipes”). No consequences were delivered for echoing or responding to the ST. After delivering the ST, the experimenter delivered one of the instructions for the PT and conducted tact teaching as described above.

#### Consequence Condition

Figure 1 (bottom panel) depicts the sequence of steps during the consequence condition. The trial procedures were the same as in the antecedent condition, except the ST was delivered *after* verbal praise (e.g., “Good job! It is a plumber! They fix pipes”). Consistent with past research (e.g., Delmolino et al., 2013), ST were delivered *only* for independent correct PT responses. In other words, if Harrison erred on or required a prompt for the PT, the ST was not provided at that time. Instead, error correction or prompting was implemented as described above. Once Harrison responded correctly to the PT (within the three repetitions), the experimenter delivered the ST following the praise (e.g., “You’re right! It is a plumber. They fix pipes”). If no independent, correct attempts were emitted within three trials, the experimenter moved on to the next trial.

#### Target Probes

Once Harrison reached mastery criteria for a PT set, the experimenter probed the corresponding ST. ST probes were conducted as described during baseline. If ST mastery was not initially demonstrated, the experimenter conducted three more trial blocks of each teaching condition^1^, repeated the ST probes, and continued this sequence until mastery of the ST was obtained^2^.

##### Intraverbal Probes

Starting in phase 2, once mastery of the ST occurred, we conducted intraverbal probes based on the ST and PT (i.e., in the absence of the picture cards). The experimenter randomized the trials within blocks and asked questions such as, “What do you use a [PT] for?”, or “What do you use to [ST]?” Correct responses included the ST for PT intraverbals (e.g., “What do you use a [sink] for?” “to wash hands”) or the PT for ST intraverbals (e.g., “What do you use to [wash hands]?” “sink”). No reinforcement or prompts were programmed. In phase 3 only, intraverbal probes were conducted during baseline to compare performance to post-teaching.

#### Best Teaching Condition

The type of trial configuration in which Harrison reached ST mastery first was used to teach the ST for the control set in phase 1. In other words, the intervention condition that Harrison met ST mastery criteria before the other intervention condition was considered the “best intervention” and replicated to teach the control condition target set.

#### Maintenance Probes

All phases incorporated maintenance probes at the end of teaching. In phase 1, maintenance probes were conducted 1 month after mastery of PT and ST were observed. In phase 2, we completed maintenance probes 1 week and then 1 month after mastery of antecedent and consequence intraverbals were observed. Finally, in phase 3, we completed maintenance 1 week and 1 month after mastery of either the consequence ST or the antecedent intraverbal was observed. The trial arrangement and procedures for maintenance probes were identical to the baseline condition.

#### Phase Specifications

Some minor procedural differences were present across phases as illustrated in Table 2 and described below.

**Table 2:** Phase differences across the three phases

Specific change	Phases		
	Phase 1	Phase 2	Phase 3
Trial blocks	5-trial block	15-trial blocks	15-trial blocks
Mastery criteria	PT – 4/5 (80%) + for 3 consecutive blocks ST – 4/5 (80%) + for 1 block	PT – 12/15 (80%) + for 3 consecutive blocks ST – 12/15 (80%) + for 3 consecutive blocks	PT – 12/15 (80%) for 3 consecutive blocks ST – 12/15 (80%) for 3 consecutive blocks
Control condition	N/A PT only (control)	Intraverbal – 80%+ for 1 block N/A	Intraverbal – 80%+ for 1 block N/A
Intraverbal probes	N/A	Completed	Completed with baseline probes
Maintenance probes	1-month maintenance probes	1-week and 1-month maintenance probes	1-week and 1-month maintenance probes
Condition S^D^s	N/A	N/A	Blue and yellow correlated stimuli added to each condition
Concurrent-chains condition	N/A	N/A	Added to measure differential responding toward one condition over another

*PT*: Primary target; *ST*: Secondary Target; *N/A*: not applicable; *S*^*D*^: discriminative stimuli

##### Phase 1

During phase 1, we incorporated the PT-only condition to serve as a control for the conditions that included ST. Sessions were also distinct from the other phases based on the number of trials in a block (5) and the mastery criteria for the ST (one block at 80% or more [4/5] independent correct responses).

##### Phase 2

During phase 2, we incorporated intraverbal probes after mastery of the PT and ST were observed to assess for the transfer-of-stimulus control from PT and ST tacts to intraverbals. Sessions consisted of 15-trial blocks with mastery criteria set at three consecutive blocks at 80% or more (12/15) independent correct responses for both PT and ST. Mastery for the intraverbal response was one block at 80% or higher.

##### Phase 3

During phase 3, we added intraverbal probes during baseline so that we could compare responding prior to intervention and post-intervention. We also added color-correlated stimuli to each ST condition (i.e., blue and yellow cards) to help enhance Harrison’s discrimination across conditions. Sessions and mastery criteria were the same as in phase 2 for PT, ST, and intraverbal probes.

##### Concurrent-Chains Procedure

At the end of phase 3, we added a concurrent-chains procedure to assess Harrison’s preference for the delivery of the ST. Targets from both conditions were quasi-randomly mixed and counterbalanced in three, 10-trial blocks to control for potential confounds related to stimulus/item biases and decrease the probability of order effects. The trial started by presenting the yellow and blue cards and asking, “Do you want to do the picture with the yellow or the blue square?” Harrison could point to or vocalize his response. Harrison then experienced a typical tact-teaching trial with the ST delivery in the antecedent or consequence portion of the trial based on his color selection. For example, if Harrison selected yellow, the experimenter would conduct one trial placing the ST in the antecedent portion of the trial; if Harrison selected blue, the experimenter would deliver the ST in the consequence portion of the trial. We then compared the differential selection of one condition over the other, suggesting a possible preference for that ST configuration (Auten et al., 2024).

## Results

### PT and ST Acquisition Based on ST Locus

**Fig. 2:**
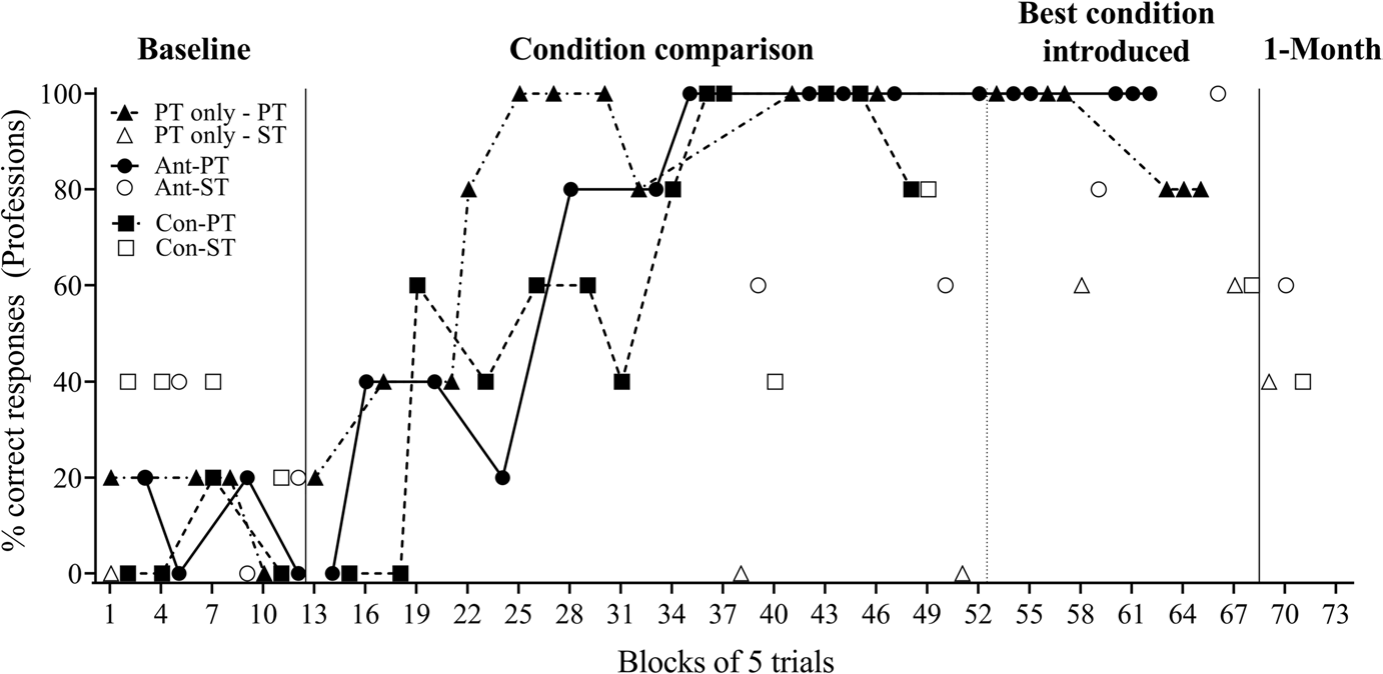
Percentage of correct responses for antecedent, consequence, and PT-only (control) conditions for phase 1 (professions). *Note*. Primary target (PT) and secondary target (ST) for antecedent (ant), consequence (con), and PT-only (control) across baseline, intervention, and 1-month (maintenance test)

Figure 2 shows the percentage of correct responses for all conditions for the PT and ST for phase 1. During baseline, the percentage of correct PT responses across all conditions was below 20% and at or below 40% for ST. During intervention, regardless of the ST locus, the percentage of correct PT responses increased gradually until mastery criterion was observed. Harrison first reached mastery for ST in the consequence condition; the antecedent condition had 60% independent responses for the ST, and no correct ST responses were observed in the PT-only condition. As such, we taught the PT-only (control) set using consequence ST (block 53). Levels of correct ST responses increased from 0% to 60% after three blocks of teaching. Harrison also reached mastery for the antecedent ST, which increased to 100% while his consequence-ST responses decreased to 60%. One-month maintenance probes showed a slightly higher percentage of correct ST responses for the antecedent condition (60%) compared to both the consequence and PT-only conditions (40%).

Table 3 displays the results for prompts and error corrections across conditions for each phase. In phase 1, Harrison required 16 prompts in the consequence condition compared to 10 and eight prompts in the antecedent and PT-only conditions, respectively. However, Harrison required eight error corrections in the PT-only condition and six error corrections in both antecedent and consequence conditions.

**Table 3:** Efficiency measures for antecedent, consequence, and PT-only conditions for all three phases

Intervention Variables				Trials-to-criterion^ab^	
Phase	Condition	Prompts	Error correction	Primary targets	Secondary targets
1	Antecedent	10	6	35	**65**
	Consequence	16	6	50	**65**
	PT only (control)	8	8	**30**	No mastery
2	Antecedent	7	2	**45**	135
	Consequence	8	3	75	**75**
3	Antecedent	7	21	**75**	165
	Consequence	9	21	120	**135**

*PT*: Primary target; Bold indicates the most efficient condition for that phase/target. ^a^ Phase 1 is 5-trial blocks only; ^b^ Phases 2 and 3 are 15-trial blocks

**Fig. 3:**
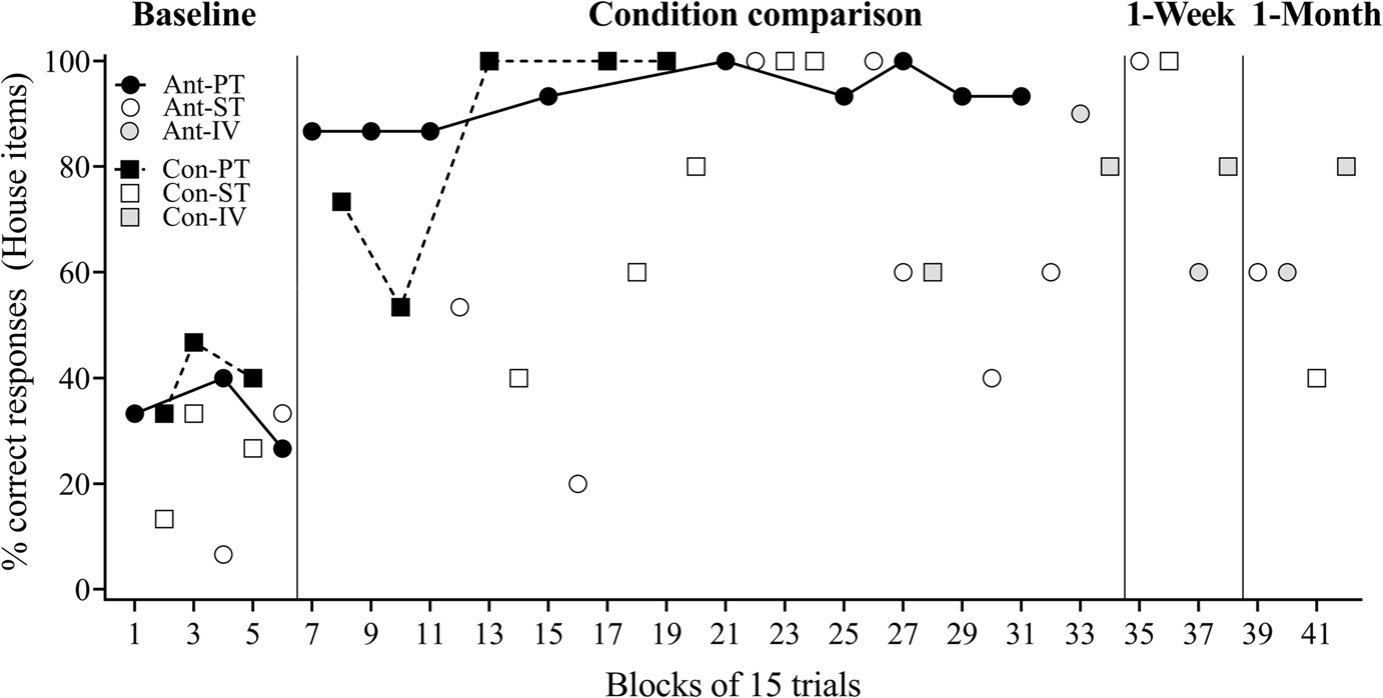
Percentage of correct responses for antecedent and consequence conditions for phase 2 (house items). *Note*. Primary target (PT), Secondary target (ST), and intraverbals (IV) for the antecedent (ant) and consequence (con) conditions across baseline, intervention, 1-week, and 1-month (maintenance tests)

Figure 3 shows the percentage of correct responses for the PT, ST, intraverbals, and in maintenance for phase 2. During baseline, correct responses for PT and ST across both conditions were less than 50%. Once the intervention began, both procedures produced an immediate increase in correct responses for the PT and a gradual increase for the ST responses. ST acquisition in the consequence condition had a linear increasing trend until mastery was observed, while acquisition in the antecedent condition was variable. After two trial blocks at 100% for the ST in the antecedent condition, Harrison’s scores subsequently decreased. However, due to high scores on the antecedent intraverbal probe (90%), which was the terminal response, we ceased further PT teaching and ST probes in the antecedent condition (i.e., implemented a nonparallel stopping criteria for the ST as described in the method section).

One-week maintenance probes show 100% correct responses for both ST. One-month maintenance probes show a slightly higher percentage of ST correct responses for the antecedent condition (60%) than the consequence condition (40%), but lower performance overall compared to the 1-week probe. Table 3 shows that Harrison required eight prompts in the consequence condition compared to seven prompts in the antecedent condition. Harrison required three error corrections in the consequence condition compared to two error corrections in the antecedent condition.

**Fig. 4:**
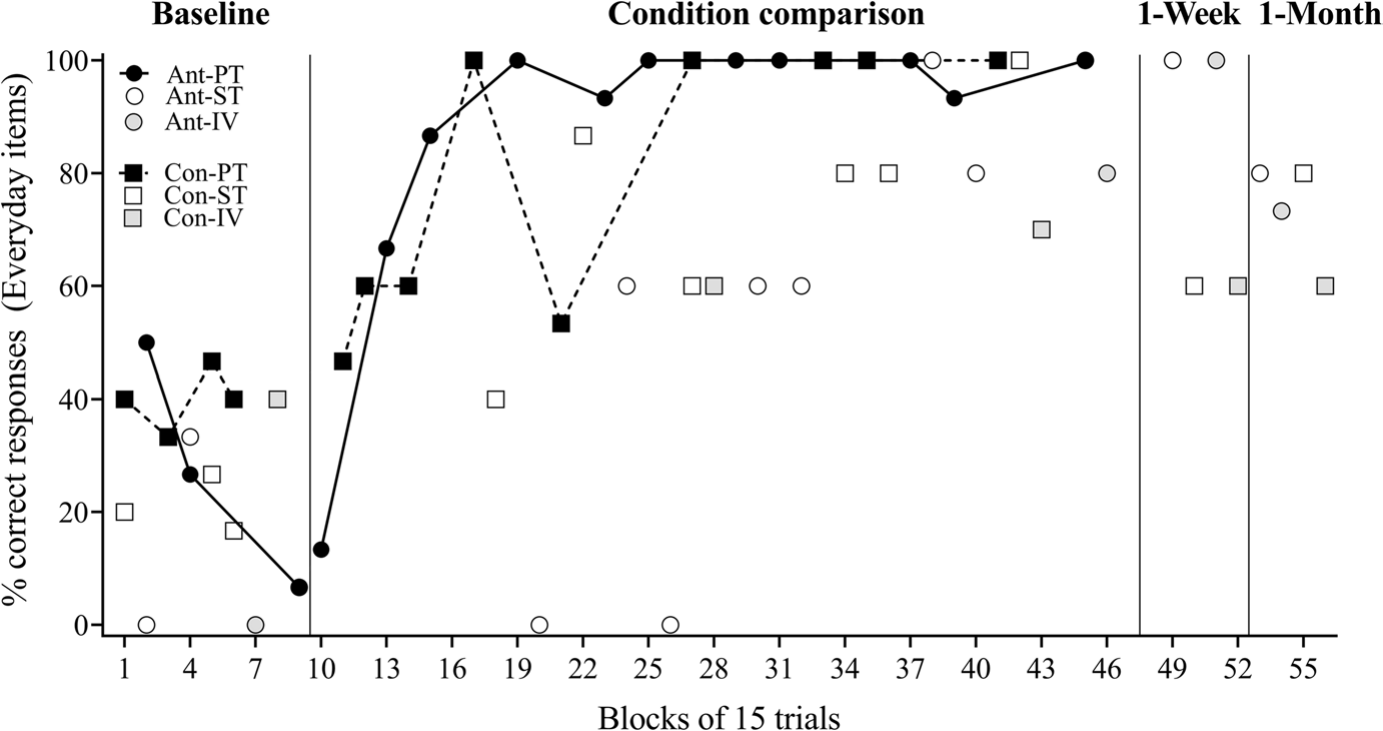
Percentage of correct responses for antecedent and consequence conditions for phase 3 (everyday items). *Note*. Primary target (PT), secondary target (ST), and intraverbals (IV) for the antecedent (ant) and consequence (con) conditions across baseline, intervention, 1-week, and 1-month (maintenance tests)

Figure 4 shows the percentage of correct PT and ST responses for antecedent and consequence conditions for phase 3. During baseline, the percentage of correct PT responses in the antecedent condition decreased from 50% to 7%, while responding in the consequence condition remained around 40%. The percentage of correct ST responses was lower than 33% in both conditions. Harrison met mastery for the PT for both conditions, and mastery for the consequence ST. At 1-week maintenance, Harrison’s ST responses were 100% correct during the antecedent condition and 60% correct during the consequence condition. At 1-month probes, Harrison emitted 80% correct ST responses for both conditions, which was higher than his ST responses at 1-month probes from both previous phases. Table 3 shows that Harrison required nine and seven prompts in the consequence and antecedent conditions, respectively, and required 21 error corrections for both conditions.

### Transfer-of-Stimulus Control from Tacts to Intraverbals

Figure 3 depicts Harrison’s correct PT, ST, and intraverbal responses for phase 2. Once Harrison met mastery in the consequence condition, we then probed for intraverbal responses for both conditions. Harrison scored 60% and 80% on the intraverbals from the consequence condition. Harrison scored 90% on the intraverbals from the antecedent condition despite not achieving mastery on the ST for that condition. Given Harrison met the mastery criterion, we did not continue conducting antecedent-ST trials. One-week maintenance probes showed slightly lower responding for the consequence-condition intraverbal (80%) and 60% for the antecedent-condition intraverbal compared to the ST 1-week probes, but intraverbal responding remained stable at the 1-month maintenance probe.

Figure 4 depicts Harrison’s correct PT, ST, and intraverbal responses for phase 3. Intraverbal responding in baseline was at 0% for the antecedent condition, but higher (40%) in the consequence condition. After mastery of the consequence ST, Harrison reached mastery for the intraverbal response in the antecedent condition at 80% correct responses, but the intraverbal responses in the consequence condition remained below mastery. At 1-week maintenance, Harrison’s intraverbal responses were 100% correct for the antecedent condition and 60% correct for the consequence condition. The 1-month intraverbal maintenance probes showed a decrease in responding for the antecedent condition at 73% correct responses, while the consequence condition remained stable at 60%.

### Intervention Efficacy

**Fig. 5:**
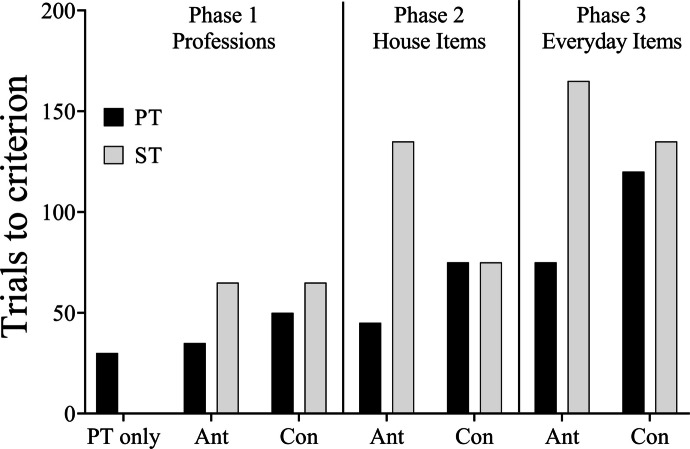
Comparison of trials to criterion for PT and ST across conditions for all three phases. *Note*. Primary target (PT), secondary target (ST) for the antecedent (ant) and consequence (con) conditions across all three phases. *ST did not meet the mastery criterion this condition

To measure intervention efficacy, we compared trials-to-criterion for PT and ST (Table 3). Figure 5 shows the aggregated differences across conditions. In phase 1, Harrison required 35 trials in the antecedent condition, 50 trials in the consequence condition, and 30 trials in the PT-only condition to reach mastery for the PT. Harrison required 65 trials to reach mastery for the ST in both antecedent and consequence conditions. In phase 2, Harrison required 45 trials to reach mastery for PT in the antecedent condition and 75 trials in the consequence condition. In phase 2, mastery of ST was achieved in 75 trials during the consequence condition. Harrison did not reach mastery of the ST in the antecedent condition. Despite that, Harrison demonstrated mastery of the antecedent ST during intraverbal probes (90%), an arguably more complex skill, after 135 trials. Therefore, teaching trials of the antecedent ST ceased, creating a nonparallel stopping criteria for the ST across antecedent and consequence conditions in phase 2. In phase 3, Harrison required 75 trials to reach mastery of the PT in the antecedent condition and 120 trials in the consequence condition. Mastery of ST was achieved in the antecedent condition in 165 trials and in 135 trials in the consequence condition.

The outcomes of phase 3 reproduced phase 2’s findings for both PT and ST acquisition. Similarly, results of phase 1 show that fewer trials were needed for the PT in the antecedent condition compared to the consequence condition, although the PT-only condition required the least number of trials. Both the antecedent and consequence conditions required equal numbers of trials before mastery of the ST. Across all three phases, more trials were needed for Harrison to reach PT mastery criteria in the consequence condition than in the antecedent condition. However, for phases 2 and 3, more trials were needed to reach ST mastery in the antecedent condition than in the consequence condition. Harrison required 24 prompts and 29 error corrections for the antecedent condition, and 33 prompts and 30 error corrections for the consequence condition, across all three phases and conditions.

### Preference for the ST Locus

**Fig. 6:**
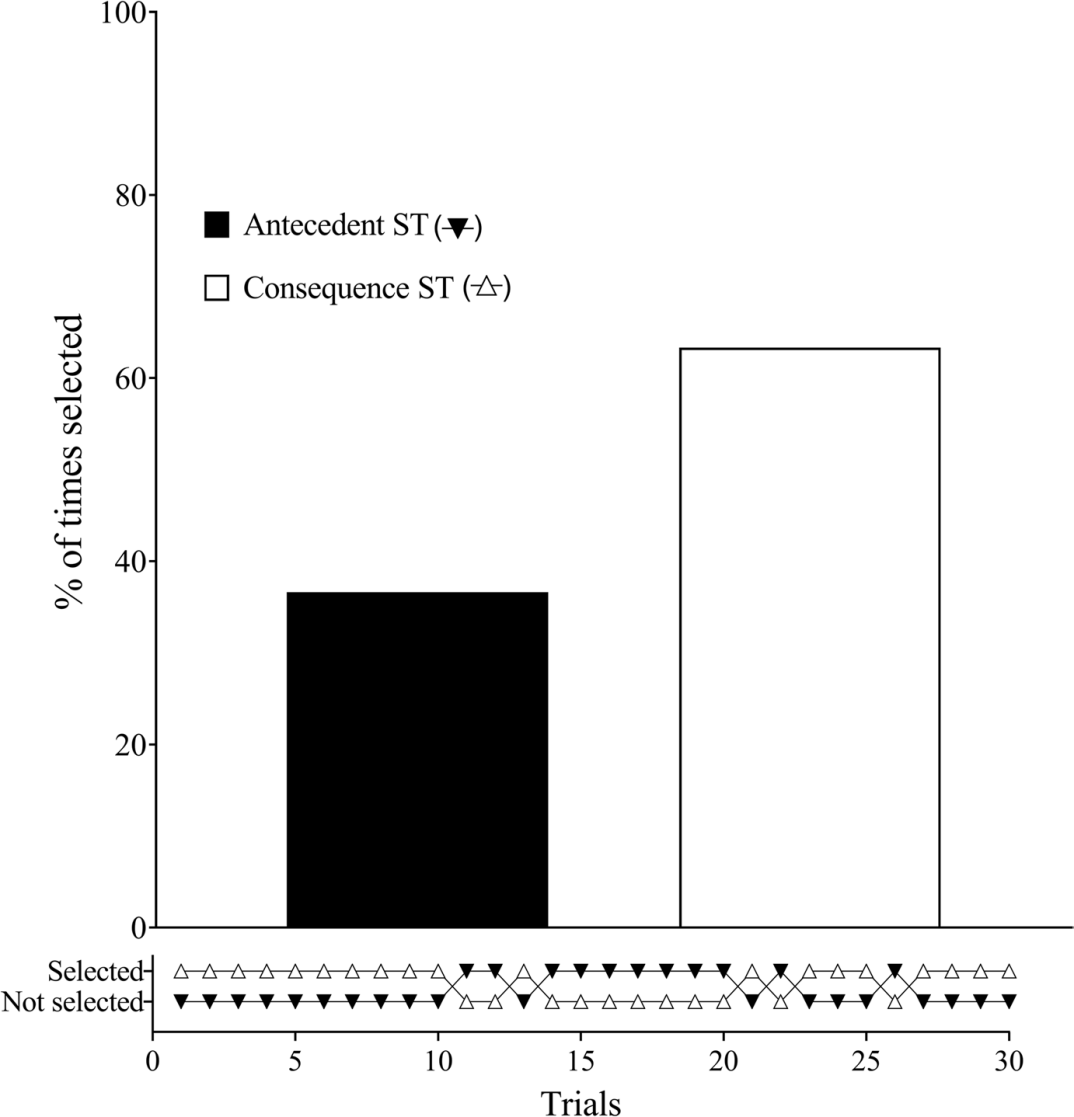
Overall percentage and trial-by-trial choice of antecedent or consequence ST during the concurrent-chains condition. *Note*. The top figure shows the overall percentage of choice of discriminative stimuli (yellow and blue cards) correlated with antecedent- and consequence-ST conditions. The bottom part of the figure shows the trial-by-trial selection of each stimulus correlated with the ST conditions across 30 trials

Figure 6 shows the aggregated condition selection data from the concurrent-chains procedure and trial-by-trial selections. Overall, Harrison selected the consequence-ST link on 63.3% of trials and the antecedent-ST link on 36.7% of trials. When examining the trial-by-trial pattern (bottom of Fig. 6), during the first 10-trial block, Harrison exclusively selected the consequence link (100%). During the second trial block, Harrison shifted to almost exclusively selecting the antecedent cue (90%). Finally, in the last trial block, Harrison alternated between both cues initially before almost exclusively choosing the consequence cue (80%).

## Discussion

Six findings related to differences in efficacy and efficiency of delivering the ST in the antecedent or consequence locus of the trial across the three phases were identified in the current study:

- PT acquisition was more efficient (i.e., fewer trials to achieve mastery) when ST were delivered as an antecedent as compared to as a consequence;
- ST acquisition was more efficient when ST were delivered as a consequence as compared to an antecedent;
- Providing ST in any locus of the trial lead to ST acquisition as compared to no ST at all;
- No consistent differences were observed in ST maintenance between conditions; however, there seemed to be slightly better maintenance in the antecedent condition at both 1-week and 1-month probes;
- Intraverbal acquisition seemed to be marginally more efficient in the antecedent condition than the consequence condition, but maintenance of intraverbal responses across conditions was mixed; and
- Harrison chose the condition that delivered ST as a consequence slightly more than the condition that delivered ST as an antecedent.

### PT and ST Acquisition Based on the ST Locus

The first contribution of our study was that PT acquisition was more efficient when ST were delivered as antecedents rather than consequences. This effect of the ST locus on PT acquisition could be explained by stronger discrimination of the S^D^-PT reinforcer relation and history of conditional responding. In the antecedent condition, the ST stimuli may have acquired a discriminative function that set the occasion for, “What is this?” ➔ “PT” ➔ praise contingency (Wolery et al., 2000). Discriminative stimuli have been shown to establish discriminative control of three-term contingencies as a contingency-specifying stimulus (Akers et al., 2018; Blakely & Schlinger, 1987; Galizio, 1979; Saini et al., 2016; Schlinger & Blakely, 1987; Sidman et al., 1986). The ST possibly being established as an S^D^ could have enhanced Harrison’s discrimination of the DTI contingency in the antecedent condition. Conversely, in the consequence condition, the ST may have acquired a conditioned reinforcer function after repeated pairings with the reinforcer. This condition configuration could also be compatible with accounts of backward-conditioning procedures where a conditioned stimulus (e.g., “Good job, it is a plumber!”) is followed by a neutral stimulus (e.g., “They fix pipes;” Prével et al., 2016; Prével & Krebs, 2021; Spetch et al., 1981). However, this configuration could contribute to inefficient learning as the ST may have masked the PT-reinforcer relation (i.e., presentation of the ST makes the reinforcer less salient by introducing “noise;” Thomas et al., 1970, 1971). This masking effect could disrupt the participant’s discrimination of the PT-reinforcer contingency. Although demonstrations of masking or salience functions of both PT and ST are not possible in this study, a slightly higher number of prompts and error corrections in the consequence condition compared to the antecedent condition before the acquisition of the PT also supports this idea (Table 3).

A second possible explanation is a carry-over effect from Harrison’s history of reinforcement with procedures that involve conditional responding before and across the three phases. Several studies have reported the effects of the history of conditional responding on the emergence of conditional relations (Leader et al., 2000; Minster et al., 2011; Nartey et al., 2015; Nedelcu et al., 2015; Smeets & Barnes, 1997). As it was a within-subject replication, Harrison was repeatedly exposed to the same structured format, which could have increased the probability of acquisition with future novel stimuli. This has been studied as an unreinforced conditional selection of comparison stimuli (Saunders et al., 1988), where novel stimuli presented at the same spatiotemporal arrangement acquire functions of previously established conditional discriminations (Fields & Arntzen, 2018; Minster et al., 2011; Nartey et al., 2015; Nedelcu et al., 2015; Saunders et al., 1988; Williams et al., 1995).

Acquisition of ST was marginally more efficient when presented as a consequence rather than an antecedent but delivering ST in either locus was better than providing no ST at all (Vladescu & Kodak, 2013). Consistent with past research, incorporating the ST in the consequence portion of the trial may increase the saliency of the ST-reinforcer pairing as the ST is close to the reinforcer delivery, therefore, potentially increasing its saliency (Tullis et al., 2018). Similarly to our findings, research that has compared both types of ST loci has found inconsistent or minimal differences in ST acquisition (Haq et al., 2017; Vladescu & Kodak, 2013; Wolery et al., 2000), suggesting the ST locus is irrelevant or may be idiosyncratic, possibly related to the learner’s response characteristics (Kodak et al., 2011) or instructional history (Coon & Miguel, 2012).

### Transfer-of-Stimulus Control from Tacts to Intraverbals

Despite more efficient ST acquisition when ST were delivered as consequences over antecedents, another contribution of our study is that we found slightly higher acquisition of intraverbals in the antecedent condition. Close temporal contiguity between different covert tacts (i.e., name and function) of the nonverbal stimulus, may account for the emergent intraverbal responses (Frampton & Shillingsburg, 2020; Palmer, 2016; Skinner, 1957). Some researchers have suggested that an intraverbal bidirectional naming repertoire might be relevant to ST acquisition as a higher-order operant where the participant establishes intraverbal relations as related or equivalent (Horne & Lowe, 1996; Miguel, 2018). In the antecedent condition, Harrison repeatedly heard the experimenter use the ST and PT contiguously (e.g., wash hands and sink). Harrison may have then repeatedly self-echoed this relation such that it became a bidirectional relation (e.g., wash hands and sink). Then, when he was asked (in the absence of the picture), “What do you use to (wash hands)?” or “What do you use a (sink) for?”, the auditory products of “sink” or “wash hands” would evoke the corresponding intraverbal response. It could be that the strength of the bidirectional relation in the antecedent condition was slightly higher compared to the consequence condition because the ST-PT relation was reinforced with praise, rather than preceded by praise, due to its locus in the trial. Unfortunately, in the absence of data on Harrison’s echoic behavior during the study, this remains an area in need of further investigation. Though the current conceptualization of learner characteristics orients readers toward a generalized echoic repertoire, it does not explicitly elaborate on the various learner characteristics that may be responsible for acquiring untrained targets (Shawler et al., 2023).

### Preference for the ST Locus

Another contribution of the current study involves Harrison’s responding during the concurrent-chains procedure. To our knowledge, our study is one of the first attempts to identify a preference when using IF (c.f. Anderson & Wiskow, 2025), and the first to assess preference for the type of ST locus within teaching. As is becoming more common in behavioral literature (e.g., Auten et al., 2024), when two or more procedures result in similar intervention efficacy, an important consideration should then include the learner’s intervention preference. Harrison allocated a higher proportion of responses to the cue that signaled the delivery of ST as a consequence over antecedent, which could suggest a slight preference for the consequence configuration. Consistent with research on concurrent-chains procedures (Auten et al., 2024; Owen et al., 2021), allocating responding toward one correlated signal over another could be indicative of a preference for the procedure following that signal. However, given only two options to select, it is plausible that Harrison’s selection may have been under the control of other variables than preference (e.g., color bias, conditional discrimination issues).

Furthermore, preference was measured as the relative frequency of responses allocated to the initial link (of each chain) in the concurrent-chains procedure (Lockhart, 1979). This measure is an indirect assessment of preference (Lockhart, 1979), specifically because selection responses may not have resulted in differential reinforcement of higher rates of responding to the *initial* link (Lockhart, 1979). In this case, reinforcement was contingent upon responding to the *terminal* link in the concurrent-chains procedure.

Upon closer investigation, Harrison’s first 10 selections demonstrate an exclusive preference for the consequence condition. However, when considering all of his selections, some variability in preference is observed on the basis of the switches in selection responses over time. Auten et al. (2024) recently found variability across published studies in pre-experimentally established criteria for determining preference. For example, Owen et al. (2021) reported a preference after six consecutive selections of the same condition. Alternatively, Argueta et al. (2019) determined a preference if participants selected one option four more times than the other, and Jessel et al. (2020) considered a preference if a participant selected a given condition three consecutive times.

Response allocation or preference is a dynamic process that changes along with the contingencies operating on an organism’s behavior. As discussed previously, numerous variables may have influenced a slightly disproportionate allocation of responses across the two interventions. For instance, delivering the ST as a consequence was more closely tailored toward Harrison’s learning history with traditional DTI (Coon & Miguel, 2012; Vladescu & Kodak, 2013). The ST provided as a consequence may also have been preferred as it was a more efficient and effective procedure for Harrison, albeit minimally so (Goldman et al., 2024).

## Limitations and Future Research

In addition to the limitations mentioned previously, additional limitations of the current study are noteworthy. First, past IF studies have found a relationship between the frequency of attending and echoic behavior on the acquisition of ST (Haq et al., 2017). Haq et al. (2017) suggested that the efficiency and efficacy of different IF configurations could depend on the frequency of attending and echoic behavior. Future research should collect data on attending and echoic responding to further support these possible correlations. Similarly, one or more of a multitude of variables may influence the acquisition of ST. Contributions from this study suggest that competing responses when the ST was delivered, such as disruptive behavior, could explain the more efficient acquisition of ST during the consequence condition. Specifically, Harrison would sometimes try to negotiate for more time or change the topic when trials began, which may have competed with attending and possibly echoing during the antecedent condition as compared to the consequence condition. Future research must focus on pre-existing learning histories when considering various interventions such as assessing for the presence of bidirectional naming or echoic repertoires, which may shed some light on the behavioral mechanisms involved in emerging responses (Shawler et al., 2023). Thus, more within- and between-subject replications and measurement of these responses, specifically, are necessary to better understand this relationship.

Second, despite probing and identifying targets that had minimal or no responding at the start of the study, Harrison demonstrated some independent responding across sets in each baseline phase. Each phase was conducted sequentially, so it is possible that once phases 2 and 3 started, Harrison had some contact with and learned the PT or ST, as they were common or everyday items. Future research should probe possible targets before starting each respective phase (rather than at the beginning of the study) to decrease the likelihood of selecting known targets.

Third, overall differences in baseline across phases suggest the possibility that some sets could have been more “difficult” to learn than others. We conducted a logical analysis of the PT and ST sets across conditions, counterbalancing the number of syllables and words (following the guidelines by Cariveau et al., 2020). However, it is unclear if the number of syllables or words inherently controls the difficulty of learning a target (i.e., number of trials). For example, a cumulative response analysis of PT and ST in phase 1 shows that some ST were acquired after two probes, others needed six to eight probes, and two ST were never acquired. Although no differences in percentage of correct responses were observed in baseline across conditions in the three phases, future studies could improve conclusions by implementing a molecular analysis of target acquisition as appropriate.

Fourth, using a single participant to replicate the intervention effects across the three phases might introduce a limitation that weakens the overall strength of our conclusions. Although our study was focused on conducting a within-subject comparison rather than a between-subjects comparison to address one of the limitations previously reported in the literature (Haq et al., 2017; Tullis et al., 2018; Vladescu & Kodak, 2013), using a design such as a multiple-baseline design across the three phases may have been challenging due to the study’s comparative purpose. Additionally, antecedent and consequence conditions were not counterbalanced across phases; however, conditions were randomly alternated across days such that there was no consistency in which session occurred first. Future research should replicate this study using a more stringent design, such as a multiple-baseline design across sets, with multiple participants, including counterbalancing across ST conditions and controlling for the number of ST exposures to evaluate the replicability and generality of our findings more rigorously.

Fifth, consistent with past research (e.g., Delmolino et al., 2013), we only delivered the ST during the consequence condition following a correct response. Conversely, in the antecedent condition, the ST was delivered at the start of every trial. Thus, the number of prompts and error corrections required in the consequence condition could suggest an imbalance in terms of exposure to the ST. However, although we report the number of prompts and error corrections required, these data do not reflect whether Harrison eventually responded correctly within the trial, and therefore, contacted the ST. As such, these data likely misrepresent the exposure to the ST in the consequence condition to some degree. Nevertheless, we still found that Harrison learned the ST more efficiently during the consequence condition. Future research should better control for and record differences in exposure to the ST across conditions to mitigate potential differences these effects may have on the ST acquisition (Nottingham et al., 2020).

Finally, in phase 3, it is possible that Harrison’s choice behavior was controlled by the initial link characteristics (i.e., color) or the specific rule (“Do you want to do the pictures with the yellow or the blue card?”), rather than the ST locus, as we did not assess for other variables such as specific color preference (c.f. Owen et al., 2021). Future studies could replicate the concurrent-chains procedure with either modified instructions or arrangements, including a control option, which could also potentially function as a method of measuring assent for participation (Morris et al., 2021, 2024).

## Conclusion

Our study shows that the acquisition and efficiency of PT and ST were generally reproduced across the three phases for the same participant. Embedding ST in the consequence locus of the trial was slightly more efficient for ST acquisition but less efficient for PT acquisition than embedding ST in the antecedent portion of the trial. We also found that Harrison selected the initial link to access ST as a consequence more during the concurrent-chains procedure, which aligns with the slightly more efficient procedure. The efficiency of IF procedures may depend on an interaction of learner characteristics and learner history with processes related to conditional discrimination (e.g., condition-correlated stimuli at the initial link). Future research could build on this study’s contributions by isolating specific components of the learner’s conditional discrimination history or parameters related to the efficacy of ST acquisition (e.g., duration or intensity of the conditioned stimuli in backward-conditioning) or ST preference (e.g., the role of condition-corelated stimuli at the initial link). Identifying possible behavioral mechanisms may lead to more efficient and individualized instructional strategies for learners who may benefit from prompt-based or function-based teaching procedures.

## Supplementary Information

Below is the link to the electronic supplementary material.

Supplementary file1 (DOCX 15 KB)

## Data Availability

Data available upon reasonable request from the corresponding author.

## Footnotes

1 Note, due to a procedural error, only two trial blocks of the control condition were conducted prior to the consequence ST meeting mastery.

2 One exception was made in the antecedent condition in phase 2 as a nonparallel stopping criterion was implemented as described in the mastery criteria above.
