# Supplementary material for: Effects of Antecedent and Consequence Secondary Targets on Intraverbal Teaching
Source: Anal Verbal Behav. 2026 Jun 8;42(1):1–25. doi: 10.1007/s40616-026-00229-1 (PMC13469018; doi:10.1007/s40616-026-00229-1)
Supplement: Supplementary file 1 [file 40616_2026_229_MOESM1_ESM.docx]

*Logical Analysis for House items and Everyday tools targets*

|  |  | Targets | |  | Number syllables | |  | Number words | |
| --- | --- | --- | --- | --- | --- | --- | --- | --- | --- |
| Category | Sets | Primary | Secondary |  | PT | ST |  | PT | ST |
| House items | Set 1 | Bookcase | gather books |  | 2 | 3 |  | 1 | 2 |
|  |  | Sink | wash hands |  | 1 | 2 |  | 1 | 2 |
|  |  | Refrigerator | keep food cold |  | 5 | 3 |  | 1 | 3 |
|  |  | Basket | put laundry |  | 2 | 3 |  | 1 | 2 |
|  |  | Broom | sweep dirt |  | 1 | 2 |  | 1 | 2 |
|  | Set 2 | Wardrobe | store clothes |  | 2 | 2 |  | 1 | 2 |
|  |  | Stove | cook food |  | 1 | 2 |  | 1 | 2 |
|  |  | Vacuum | removes dirt |  | 2 | 3 |  | 1 | 2 |
|  |  | Trashcan | keep garbage |  | 2 | 3 |  | 1 | 2 |
|  |  | Nightstand | store items |  | 2 | 3 |  | 1 | 2 |
| Everyday Tools | Set 1 | Axe | chop trees |  | 1 | 2 |  | 1 | 2 |
|  |  | Chisel | scrap walls |  | 2 | 2 |  | 1 | 2 |
|  |  | Gum | chew gum |  | 1 | 2 |  | 1 | 2 |
|  |  | Belt | hold pants |  | 1 | 2 |  | 1 | 2 |
|  |  | Keyboard | write words |  | 2 | 2 |  | 1 | 2 |
|  | Set 2 | Umbrella | Cover from rain |  | 3 | 4 |  | 1 | 3 |
|  |  | Comb | Brush hair |  | 1 | 2 |  | 1 | 2 |
|  |  | Dishes | eat food |  | 2 | 2 |  | 1 | 2 |
|  |  | Rake | Scoop leaves |  | 1 | 2 |  | 1 | 2 |
|  |  | Lawnmower | Cut grass |  | 3 | 2 |  | 1 | 2 |
|  |  |  |  |  |  |  |  |  |  |
